# Editorial: Multilevel medical security systems and big data in healthcare: trends and developments

**DOI:** 10.3389/fpubh.2024.1516102

**Published:** 2025-01-28

**Authors:** Fei Fan, Siqin Wang

**Affiliations:** ^1^Economics and Management School, Wuhan University, Wuhan, China; ^2^Spatial Sciences Institute, University of Southern California, Los Angeles, CA, United States

**Keywords:** multilevel medical security systems, big data, healthcare, editorial, trend

The medical security system has emerged and gradually developed since the 18th century to alleviate the social problems and contradictions caused by industrialization. However, in the past development of basic medical security, we found that it suffers from a low level of security, insufficient adaptability to resident mobility, and insufficient sustainability, which calls for the development of multilevel medical security systems. The construction of multilevel medical security systems requires the support of a large amount of statistical data. The development of big data makes the collection and analysis of those necessary statistics possible and makes breakthroughs in health economics. For example, The National Health Service (NHS) in the UK constructs an allocation model based on regional differences in population, age structure, economic status, labor cost, morbidity and mortality, and makes corrections and predictions through previous data, to achieve the purpose of allocating medical insurance funds according to needs. In this context, this Research Topic aims to disclose the driving role of big data in establishing multi-level medical security and introduce new relative findings.

The distribution of medical and health resources is an important indicator for measuring the adequacy of medical insurance. Ren et al. studied healthcare resource allocation in all provinces of China from 2010 to 2021 and found the allocation of healthcare resources in China's four major zones has undergone a process of change from “unbalanced quantity to relatively balanced quantity,” but high-quality healthcare resources are highly concentrated in the eastern part of the country, and the problem of contradiction between people and doctors is prominent. It is recommended that the Internet plus healthcare technology be used to reshape the regional allocation of high-quality healthcare resources. Yu et al. analyzed the allocation of healthcare resources in Chinese centers for disease control and prevention from the perspective of population and spatial distribution, and to further explore the characteristics and influencing factors of the spatial distribution of healthcare resources.

Establishing a multilevel medical security system needs profound research on people with different characteristics. Dong focused on mobile populations and conducted studies on the impact of basic health insurance participation characteristics on the health of mobile populations and found that health insurance has a positive impact on health, public health services, and health service utilization among the mobile population, and that enrollment in local health insurance and Basic Medical Insurance for Urban Employees is more likely to be associated with higher levels of health and receive healthcare service utilization. Okechukwu et al. focused on female populations and explored the influences of Postpartum Medicaid eligibility extensions to puerperia. They evaluated associations between postpartum care visits and Medicaid insurance type and assessed effect modification by the delivery route and type of residence, and they found that women with pregnancy-related Medicaid insurance were less likely to attend postpartum visits and low-income women who lost their pregnancy-related Medicaid coverage after 60 days in Arizona experienced lower rates of postpartum care utilization. Zhou and Yang focused on poor populations and found medical insurance effectively mitigates household vulnerability to poverty and wealth inequality and made recommendations for government departments to establish health records for residents and to focus on compensating households that are on the cusp of escaping from poverty. Qiu and Zhang found that health shocks significantly increased the proportion of household spending on medical expenses and the effects were more pronounced in low-income households and those with health insurance. These findings offer strong support for the relative administrative department to promote public health, reduce the burden of medical expenses resulting from health shocks, and unlock the consumption potential.

Exploration of the influencing factors of medical and health development is necessary for a multilevel medical security system. Sun and Zhang found that donations have improved the overall medical level while widening the gap in medical security level between urban and rural areas and regions. The anomalous conclusion calls for an extension of donation policies. As the digital economy gets popular in China, Ding et al. conducted research on the impact of the digital economy on the high-quality development of the medical and health industry and found that the development of the digital economy has significantly promoted the high-quality development of the medical and health industry and has a better promotion in eastern and southern regions. Chen et al. explored the impact of regional healthcare development on medical collaborative innovation efficiency in the context of dual circulation strategy and found a significant positive spatial correlation.

It is also very important to discuss how big data can improve the medical security system from a practical perspective. Wang et al. developed a system for online assessment, which can be used to evaluate designated medical institutions in China. This has good implications for medical security system reform in other parts of China and in low and middle-income countries internationally.

In conclusion, the development of a multilevel medical security system is essential to address the limitations of traditional medical security frameworks, particularly in adapting to population mobility and ensuring sustainability. Leveraging big data is key to overcoming these challenges, as it enables the effective collection, analysis, and application of healthcare statistics. This Research Topic highlights significant advances in health economics, regional healthcare resource distribution, and the impact of healthcare policies on various populations. The above studies presented underscore the importance of using digital tools, such as big data and online systems, to enhance healthcare accessibility, efficiency, and equity, ensuring better medical security for diverse populations globally.

